# Testing a Culturally Adapted Colorectal Cancer Screening Decision Aid Among American Indians: Results from a Pre–Post Trial

**DOI:** 10.1089/heq.2019.0095

**Published:** 2020-04-01

**Authors:** Leah Frerichs, Cherry Beasley, Kim Pevia, Jan Lowery, Renée Ferrari, Ronny Bell, Dan Reuland

**Affiliations:** ^1^ Department of Health Policy and Management, Gillings School of Global Public Health, University of North Carolina at Chapel Hill, Chapel Hill, North Carolina.; ^2^ Carolina Cancer Screening Initiative, Lineberger Comprehensive Cancer Center, University of North Carolina at Chapel Hill, Chapel Hill, North Carolina.; ^3^ Department of Nursing, College of Health Sciences, University of North Carolina at Pembroke, Pembroke, North Carolina.; ^4^ K.A.P., Inner Prizes, Red Springs, North Carolina.; ^5^ American Indian Center for Health Education and Technology, Pembroke, North Carolina.; ^6^ Department of Public Health, Brody School of Medicine, East Carolina University, Greenville, North Carolina.; ^7^ Department of Medicine, School of Medicine, University of North Carolina at Chapel Hill, Chapel Hill, North Carolina.

**Keywords:** decision support techniques, intention, American Indians, multimedia

## Abstract

**Purpose:** American Indian adults have not experienced decreases in colorectal cancer (CRC) incidence and mortality observed in other races or ethnic groups and their screening rates are low. Decision aids that explain available CRC screening options are one potential strategy to promote screening. The goal of this study was to test the effect of a culturally adapted decision aid on CRC-related outcomes among American Indian adults, including screening-related knowledge, attitudes, self-efficacy, intentions, and screening modality preferences.

**Methods:** We recruited American Indian adults aged 50–75 years who were not current with CRC screening. Participants viewed a 9-min multimedia decision aid that used narrative vignettes to provide educational information about screening along with messages to address culturally specific barriers and values uncovered in formative research. We conducted a single-arm (pre–post) study and assessed screening-related outcomes at baseline and immediately after viewing the decision aid.

**Results:** Among *n*=104 participants, knowledge scores increased from a mean of 36% correct to 76% correct. Participants also had statistically significant increases in positive attitudes, perceived social norms, self-efficacy, and intent. The proportion of participants who identified a preference for a specific CRC screening modality rose from 81% identified at pre-intervention to 93% post-intervention (*p*=0.013).

**Conclusion:** Our study provides promising new findings that our culturally adapted decision aid is efficacious in educating American Indian adults about CRC screening and increases their screening intentions and ability to state modality preferences. Future research is needed to test the decision aid as a component of CRC screening interventions with American Indian adults.

## Introduction

Colorectal cancer (CRC) is a leading cause of death in the United States.^1^ It is also a leading cause of morbidity and mortality among American Indian and Alaska Native adults, with regional differences in disparities when compared with non-Hispanic white adults.^2^ Nationally, American Indians and Alaska Native adults have not experienced decreases in CRC incidence and mortality observed in other races or ethnic groups,^1^ and screening rates are disproportionately low in this population.^3^ There are multiple screening modalities recommended for adults aged 50–75 years (primarily colonoscopy and stool-based testing) that have been shown to reduce CRC incidence and mortality.^4^ In 2016, 57.1% of American Indian and Alaska Native adults met CRC screening recommended guidelines compared with 66.1% and 68.9% of African American and non-Hispanic white adults, respectively.^5^ There is a significant need to improve CRC screening among American Indian and Alaska Native adults.

American Indian and Alaska Native adults are known to have difficulty communicating about and accessing medical care generally (e.g., an underfunded health care system has often limited access to services and impacted trust in and willingness to communicate with health care providers) and have many individual- and system-level barriers to CRC screening.^6^ System-level barriers include lack of insurance, underfunded health care systems, and complex approval processes to obtain screening. Individual-level barriers to CRC screening among American Indian and Alaska Native adults include low levels of knowledge and awareness about screening options, fatalistic fears of screening, and mistrust in health care.^7–10^ Unfortunately, there has been relatively little research to develop culturally appropriate interventions to help overcome barriers.

Multimedia patient education tools (i.e., tools that combine content such as text, audio, and video) have the potential to improve communication about CRC screening for vulnerable populations such as American Indian and Alaska Native adults. Multimedia formats can be particularly helpful to convey complicated information using graphics,^11^ and narratives from individuals from the same population can provide messages in a relatable format that have potential to engage and comfort patients.^12^ A specific approach is to use the multimedia tool as a decision aid, which in the case of CRC can help patients understand their screening options and help them determine their preferences. Research studies have shown that multimedia decision aids can improve CRC screening knowledge and intent to obtain screening and may increase screening test completion among majority non-Hispanic white,^13^ majority African American,^14–16^ and Latinx populations.^17^ However, to our knowledge, no studies have evaluated multimedia CRC screening educational interventions with American Indian and Alaska Native adults. Furthermore, communication theories suggest that individuals will be more attentive and accepting of messages that are tailored to their preferences and values, yet existing decision aids have not been tailored for American Indian and Alaska Native adults.^18^

We previously conducted a series of focus groups with American Indian adults in the eastern United States and used the findings to culturally adapt an existing CRC screening decision aid for this population.^19^ The existing decision aid was shown to be effective at improving readiness for screening among a majority African American patient population,^11^ and a culturally and linguistically adapted version significantly improved knowledge and screening intentions among Latinx patients with limited English proficiency.^17^ The objective of this study was to evaluate the impact of the CRC screening decision aid adapted for American Indian adults on their CRC-related outcomes, including knowledge, attitudes, self-efficacy, intentions, and screening modality preferences.

## Methods

We used a single-group pre- and post-intervention design to pilot test the culturally adapted decision aid. The study was approved by the Institutional Review Board at the University of North Carolina at Chapel Hill and the Lumbee Tribe of North Carolina.

### Participant recruitment and eligibility

We recruited a convenience sample from both the community at large and from clinic registry sources. Community recruitment involved in-person and word-of-mouth recruitment through public venues, primarily churches and senior centers. Clinic registry recruitment was conducted through queries of patient registration data at one Federally Qualified Health Center in Robeson County, North Carolina. The queries identified American Indian adults aged 50–75 years who were then recruited using a mailing and follow-up telephone calls. To be eligible for the study, the participants had to be 50–75 years old, self-identify as American Indian, have average risk for CRC (i.e., have no personal history of precancerous polyps or first degree family history of CRC), and not be current with CRC screening, defined as having a colonoscopy within 10 years or a fecal occult blood test (FOBT)/fecal immunochemical test (FIT) within the past year. Participants also had to have the ability to provide informed consent.

### Intervention description

We developed the decision aid using a formative research process aimed at producing a cultural adaptation of a previously developed and tested decision aid.^14,20^ The original decision aid was informed by several behavioral theories including social cognitive theory and the theory of planned behavior. Our culturally adapted decision aid was further informed by cultural identity theory,^21^ which posits that one's cultural identity and corresponding values underlie how one receives and interprets communicated messages and how one communicates with others about these messages. Details of our formative research findings are reported elsewhere.^19^ In brief, our formative research uncovered themes of American Indian perspectives of CRC screening that included fear/fatalism surrounding cancer and screening, mistrust of health care providers and systems, collectivism values (stronger orientation to family and community health than individual health), and privacy and communication concerns.^19^ We used a purpose-, content-, and valence-based taxonomy^12^ to create content that addressed the uncovered themes using patient narratives and illustrative examples. The translation followed a process similar to prior cultural adaptations of the decision aid.^22^

### Decision aid content and format

**FIG. 1.**
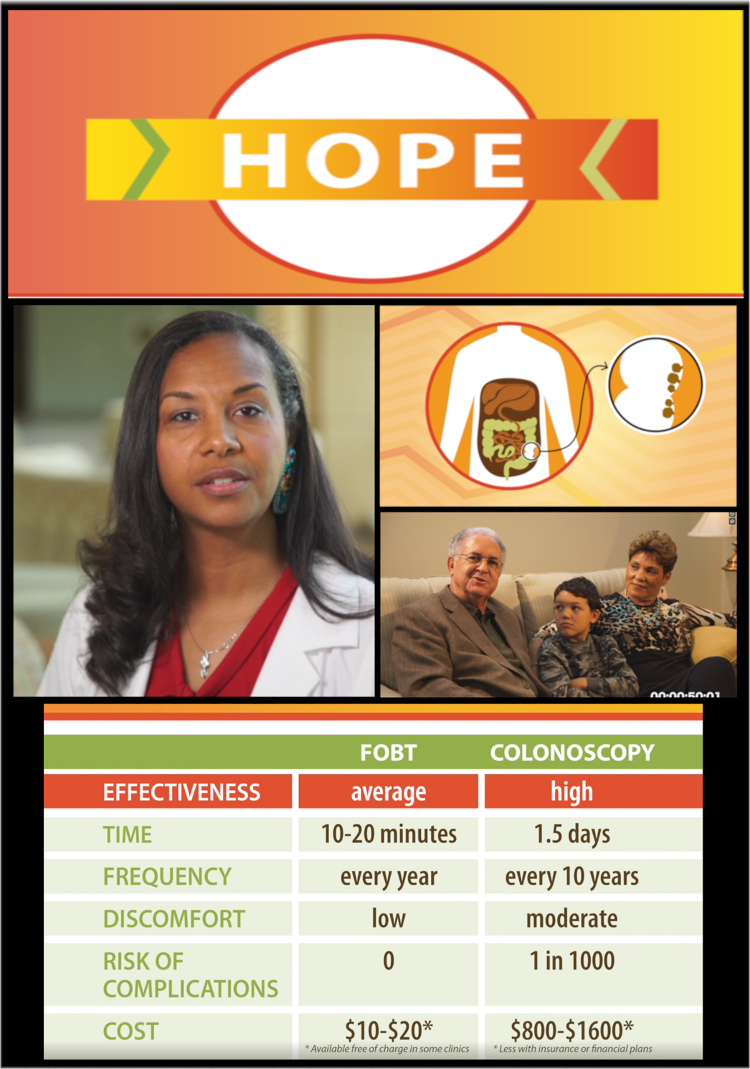
Screenshots from the decision aid adapted for American Indian adults. FOBT, fecal occult blood test.

The decision aid was a 9-min video (see Fig. 1 for screenshots). The video could be viewed through web streaming or downloaded on an electronic device. The content included an overview and rationale for CRC screening, and specific information about and comparisons of two major testing modalities: colonoscopy and FIT/FOBT, which was all narrated by a leader from a tribal community. There were also narrative vignettes from the following: a faith/religious leader, a gastroenterologist, a CRC survivor, and an intergenerational family, all of whom were individuals from American Indian communities in the eastern United States. The vignettes embedded educational information about screening along with messages to address the themes uncovered in the formative research. The decision aid was designed to be accessible regardless of literacy level. All written text was read aloud by a narrator, and technical terms and concepts were explained using easy-to-understand narration, vignettes, and graphics. The end of the decision aid video provided viewers with generalized information about where to obtain CRC screening and prompted the viewer to speak with a health care provider.

### Data collection

Participants were enrolled by telephone or in-person. They first completed a brief screening survey to assess for eligibility. Eligible telephone recruits were scheduled to view the decision aid at a later date. Eligible in-person recruits immediately viewed the decision aid in-person or were scheduled to do so at a later date. Decision aid viewing was not linked to clinical visits, and participants viewed the decision aid privately in their homes or at the location from which they were recruited (i.e., church or senior center). Immediately before viewing the decision aid, participants completed informed consent and a self-completed baseline questionnaire. Participants then watched the decision aid video and immediately completed a second questionnaire. Depending on their comfort level, participants could either complete questionnaires electronically on a tablet (entered directly into a database) or a pen and paper survey that was subsequently entered into a database. Data were collected from October 2017 through September 2018. Participants received a $25 gift card after the completion of the in-person decision aid viewing appointment and survey.

### Measures

#### Knowledge

Knowledge was assessed by a five-item index based on decision aid content. Items included (1) the availability of more than one option for CRC screening, (2) the availability of a home screening test, (3) the recommended age to begin CRC screening, (4) FOBT screening test frequency, and (5) the existence of a small but nonzero complication risk associated with having a colonoscopy.

Responses to the knowledge items were in a true–false format with a third response option of “don't know” offered.

#### Attitudes

Attitudes were assessed by an adapted five-item scale of “pros” and a five-item scale of “cons” (α=0.78).^23^ Participants were asked to assess how important various items were for their decision of whether or not to get CRC screening, and included potential pros (e.g., having peace of mind after receiving clear findings) and cons (e.g., being concerned that screening is painful). The items had 4-point Likert scale response options (1=“not at all true of me” to 4=“very true of me”). Both “pros” and “cons” scales had acceptable internal consistency (α=0.77 and 0.71, respectively).

#### Perceived social norms

Social norms were assessed by an adapted four-item scale^23^ of social influences that assessed the participant's belief that family, friends, and doctors think he/she should be screened. For example, participants were asked to rate the extent that they believed that “My doctor thinks I should get tested for colon cancer” on a 4-point Likert scale (1=“not at all true of me” to 4=“very true of me”). The scale had good internal consistency (α=0.80).

#### Self-efficacy

Self-efficacy was measured using an adapted four-item scale^23^ that assessed confidence to complete screening, with 4-point Likert scale response options (1=“not at all sure” to 4=“very sure”). For example, participants were asked to rate “How sure are you that you can complete colon cancer screening?” The scale had acceptable internal consistency (α=0.76).

#### CRC screening preferences

We used a measure of screening preferences from prior studies.^24^ Participants were asked, “If you had to choose a colon cancer screening test, which test would you prefer?” with multiple choice response options of the different tests (i.e., FOBT/FIT and colonoscopy) and options to indicate they had no preference or did not have enough information to decide.

#### CRC screening intentions

We used a visual analog scale to measure screening intent. Visual analog scales have been shown to be useful and valid tools to evaluate subjective characteristics and attitudes in health research.^25,26^ The scale allowed participants to choose their intent level along a bar that ranged from “not at all planning to get screened” on the left side to “definitely planning to be screened” on the right. Their placement along the scale was translated into a number that ranged from 1 to 100.

### Analysis

We used descriptive statistics (percentages) from the baseline questionnaire to characterize the study population. To evaluate impact on CRC screening knowledge, we treated “don't know” responses as incorrect responses and dichotomized the five individual knowledge item responses as either correct or incorrect. We calculated the portion of the five items that each participant answered correctly at baseline and postviewing. We then used a paired Student's *t*-test to assess for statistically significant differences in pre- and postviewing knowledge scores. We averaged the items for the attitudes, social norms, self-efficacy, and screening intentions scales and assessed for pre- to postviewing differences using a paired *t*-test. We also assessed for differences using the Wilcoxon signed rank test and found the same results. To ensure accurate measurement of these scales, we excluded participants who answered <60% (five-item scales) and 75% (four-item scales) of the items for the respective measure. Only two participants were excluded based on this threshold.

To assess for changes in CRC screening preferences, we dichotomized participant options at baseline as “no preference,” which included “no preference” and “not enough information to decide” or “any preference” as indicating a preference for either colonoscopy or FOBT/FIT. We used a McNemar's test to assess whether there was a statistically significant difference in the proportions of participants identifying a preference between the pre- and postviewing assessments. All analyses were completed in SAS Version 9.2 (SAS Institute, Inc., Cary, NC) and an α=0.05 significance level was applied.

## Results

At least one recruitment attempt was made with 390 individuals, *n*=247 individuals were never directly reached [i.e., unresponsive to phone call(s)], *n*=35 individuals did not participate, and *n*=4 were deemed ineligible due to age or CRC screening status. We recruited 104 participants whose characteristics are provided in Table 1. Most (76%) of the participants were recruited from the community at large and the remaining were recruited through the clinic registry. The participants were largely female (76%, *n*=79) and most had some form of health care insurance (85%). About half had a household income of <$30,000 per year and about half were retired.

**Table 1.** Participant Characteristics

Participant characteristics (*N*=104)	n	%
Gender (female)	79	76.0
Type of insurance^a^		
Medicaid	16	15.4
Medicare	55	52.9
Veteran's administration	6	5.8
Private	31	29.8
None	15	14.6
Marital status		
Married/living with partner	60	57.7
Separated/divorced	16	15.4
Widowed	24	23.1
Single never married	4	3.8
Employment status		
Employed full time	22	21.6
Employed part time	9	8.8
Unemployed	10	9.8
Retired	56	54.9
Other	5	4.9
Income		
<$10k per year	14	14.3
$10k to <$20k per year	27	27.6
$20k to <$30k per year	13	13.3
$30k to <$40k per year	18	18.4
>$40k per year	26	26.5

^a^ Categories are not mutually exclusive.

Pre- to postviewing scores in all measured outcomes are given in Table 2. Baseline CRC screening knowledge was relatively low, with a mean of 36% correct, which significantly increased to 76% after viewing the decision aid. Participants increased their perceptions about “pros” of screening from a mean score (standard deviation) of 3.6 (0.54) pre- to 3.9 (0.28) postdecision aid viewing. There was no statistically significant change in participants' perceptions about “cons” of screening. Participants also observed small but statistically significant increases in perceived social norms, indicating they viewed more individuals in their social networks to encourage CRC screening.

**Table 2.** Pre and Post Means and Mean Changes of Study Outcome Measures

Measure	Pre	Post	Mean change (95% confidence interval)	N	p
Knowledge	0.4	0.8	0.4 (0.3 to 0.4)	102	<0.0001
Attitude					
Pros	3.6	3.9	0.3 (0.1 to 0.4)	102	<0.0001
Cons	1.8	1.7	−0.0 (−0.1 to 0.1)	102	0.861
Perceived social norms	3.2	3.4	0.2 (0.1 to 0.4)	102	0.004
Self-efficacy	3.6	3.8	0.2 (0.1 to 0.3)	102	0.001
Screening intentions	44.0	56.6	12.5 (5.3 to 19.7)	99	0.001
Screening preference, % (*n*)					
Any preference	81.1 (77)	93.1 (94)		95	0.013
FIT/FOBT	21.1 (20)	29.7 (30)			
Colonoscopy	60.0 (57)	63.4 (64)			
Preferences changes, % (*n*)					
FIT/FOBT to colonoscopy		9.5 (9)		95	N/A
Colonoscopy to FIT/FOBT		5.3 (5)			
No preference to FIT/FOBT or colonoscopy		14.7 (14)			
Maintains FIT/FOBT or colonoscopy		62.1 (59)			
Maintains no preference		3.2 (3)			
Retracts initial preference		3.2 (3)			

FIT, fecal immunochemical test; FOBT, fecal occult blood test.

Screening intentions increased with statistical significance by a mean of 10.8 points (standard deviation=27.8) on a 100-point scale. Before viewing the decision aid, the mean score (standard deviation) was 44.0 (40.6), which increased to 56.6 (38.1) after viewing the decision aid. The proportion of participants who identified a specific CRC screening modality preference after viewing the decision aid was significantly greater than before viewing. Specifically, 81.1% identified a preference before viewing and 93.1% identified a preference after viewing. The majority (63.4%) identified colonoscopy as their preference after viewing the decision aid. Some participants (14.8%, *n*=14) switched their modality preferences after viewing the decision aid, 9.7% (*n*=9) switched their identified preference from FOBT/FIT to colonoscopy, and 5.4% (*n*=5) switched from colonoscopy to FOBT/FIT.

## Discussion

We found that viewing a CRC screening decision aid with cultural adaptations increased CRC screening knowledge, positive attitudes, perception of social norms, self-efficacy, identified preferences, and intent among American Indian adults. Our findings demonstrate that the information provided by the decision aid was accessible and compelling to this population. The decision aid may be a useful tool for communicating a relatively complex message about CRC screening to this vulnerable population.

A recent meta-analysis of 21 trials of CRC screening decision aids found strong evidence that viewing a decision aid results in improved knowledge and screening intentions.^27^ Our research adds to this evidence and provides new evidence that CRC screening decision aids are also associated with increased knowledge and intentions among American Indian adults, a population not represented in the previous research. Similar to past studies,^11,13–17,20,28–30^ we also found that most participants identified a screening modality preference postviewing and many identified a new preference. This is important because a major purpose and value of decision aids are to reduce suboptimal decision making and improve the decision-making experience, and our findings provide partial support that our decision aid aligns decisions with informed preferences.

This culturally adapted decision aid addressed barriers identified as important to the American Indian target population, including fear and communication challenges. We found that the decision aid was associated with improved positive attitudes and perceived social norms about CRC screening. We also found that the decision aid improved self-efficacy to a small degree. It is important to note, however, that other studies have found that although patients do become more informed and activated to get screened, screening completion rates often remain low because other barriers interfere with actual completion of screening tests.^11,17,31^ This is especially important for vulnerable populations such as American Indian and Alaska Native adults who are known to have additional challenges to access health care services, obtain financial assistance to cover the cost of colonoscopy, find reliable transportation to health care appointments, and general difficulties understanding home stool test and colonoscopy preparation procedures.^7,8,32–34^ Combining a decision aid with additional support such as patient navigation is a promising option to overcome these additional access barriers. A recent randomized trial found this combination highly effective with a low-income Latinx population.^35^

There are several limitations to our study. We used a one group (pre–post) design without a separate comparison group, and thus we are unable to test for effects compared with an attention-control condition or a less intensive intervention (written CRC screening information). We did not assess follow-up screening behaviors. There is evidence that intentions are predictive of screening behavior,^36^ but there are also known inconsistencies between intention and behavior.^37^ In addition, the interpretation of the psychosocial scales are abstract. For example, it is unclear how a unit increase in intentions or perceived norms translates to behavior change. We also used a convenience sample recruited from a localized area, which may limit the generalizability of our results to other American Indian and Alaska Native communities. Nonetheless, we attempted to recruit from diverse settings in the area and we observed positive effects. Finally, the self-reported outcomes are subject to social desirability bias.

## Conclusion

In sum, our study provides promising new findings that our culturally adapted decision aid is efficacious in educating American Indian adults about and increasing their intentions and preferences related to CRC screening. To address CRC inequities experienced by American Indian and Alaska Natives, there is a need for more research into the development and testing of culturally appropriate interventions. To that end, our study suggests that future research to test the decision aid with additional American Indian and Alaska Native adults is of value. The decision aid should be tested in randomized trials and assess CRC screening behaviors as an outcome. Alternatively, since evidence from randomized trials about CRC decision aid effectiveness on screening has already accumulated in several other populations,^27^ a hybrid effectiveness–implementation study design^38^ merits consideration for more rapid translation of research evidence to practice. This decision aid could be an effective component of CRC screening intervention efforts to address disparities in screening among American Indian and Alaska Native adults.

## Abbreviations Used

CRC: colorectal cancer
FIT: fecal immunochemical test
FOBT: fecal occult blood test
